# A Solid Ultra Fine Self-Nanoemulsifying Drug Delivery System (S-SNEDDS) of Deferasirox for Improved Solubility: Optimization, Characterization, and In Vitro Cytotoxicity Studies

**DOI:** 10.3390/ph13080162

**Published:** 2020-07-24

**Authors:** Alaa Alghananim, Yıldız Özalp, Burcu Mesut, Nedime Serakinci, Yıldız Özsoy, Sevgi Güngör

**Affiliations:** 1 Department of Pharmaceutical Technology, Faculty of Pharmacy, Near East University, Nicosia 99010, Cyprus; alaasami2489@gmail.com (A.A.); yildiz.ozalp@neu.edu.tr (Y.Ö.); 2 Department of Pharmaceutical Sciences, Faculty of Pharmacy, Jerash University, Jerash 26150, Jordan; 3 Department of Pharmaceutical Technology, Istanbul University, Faculty of Pharmacy, Istanbul 34116, Turkey; bmesut@istanbul.edu.tr (B.M.); yozsoy@istanbul.edu.tr (Y.Ö.); 4 Department of Medical Genetics, Faculty of Medicine, Near East University, Nicosia 99010, Cyprus; nedime.serakinci@neu.edu.tr; 5 Department of Molecular Biology and Genetics, Faculty of Art and Sciences Near East University, Nicosia 99010, Cyprus

**Keywords:** deferasirox, SNEDDS, solid SNEDDS, solid carriers, enhancement solubility, oral delivery

## Abstract

The research work was designed to develop a solid self-nanoemulsifying drug delivery system (S-SNEDDS) of deferasirox (DFX). According to the solubility studies of DFX in different components, Peceol, Kolliphor EL, and Transcutol were selected as excipients. Pseudo-ternary phase diagrams were constructed, and then SNEDDS formation assessment studies and solubility of DFX in selected SNEDDSs formulations were performed. DFX loaded SNEDDS were prepared and characterized. The optimum DFX-SNEDDS formulations were developed. The relative safety of the optimized SNEDDS formulation was examined in a human immortalized myelogenous leukemia cell line, K562 cells, using the MTT cell viability test. Cytotoxicity studies revealed more cell viability (71.44%) of DFX loaded SNEDDS compared to pure DFX (3.99%) at 40 μM. The selected DFX-SNEDDS formulation was converted into S-SNEDDS by adsorbing into porous carriers, in order to study its dissolution behavior. The in vitro drug release studies indicated that DFX release (Q5%) from S-SNEDDS solidified with Neusilin UFL2 was significantly higher (93.6 ± 0.7% within 5 min) compared with the marketed product (81.65 ± 2.10%). The overall results indicated that the S-SNEDDS formulation of DFX could have the potential to enhance the solubility of DFX, which would in turn have the potential to improve its oral bioavailability as a safe novel delivery system.

## 1. Introduction

Deferasirox (DFX) is an orally tridentate iron chelator agent that was approved by the United States Food and Drug Administration (FDA) in 2005, and the European Medicines Agency (EMA) in 2006, for chronic iron overload treatment as a result of blood transfusion in patients who are 2 years and older [1].Commercially, DFX is available on the market in three different dosage forms; EXJADE^®^, as a tablet for oral suspension (125, 250, and 500 mg), JADENU^®^, as a tablet dosage form (90, 180, and 360 mg), and Jadenu^®^ Sprinkle Granules (90, 180, and 360 mg). In the latter two commercial products of DFX, its dosage strength has been reduced by approximately 30% with a Pluronic-containing formulation [2].

DFX is relatively a lipophilic molecule (log P = 3.52). It is classified as a Class II drug, according to the Biopharmaceutics Classification System (BCS), which is characterized by its low aqueous solubility of 0.038 mg/mL at 37 °C [3] and high intestinal permeability [4]. The oral bioavailability of DFX was studied on EXJADE^®^, as a tablet for oral suspension dosage form is believed to be about 70%, due to its low solubility and first pass effect [5]. The dissolution of active drug substances is the rate limiting step for the absorption of BCS Class II compounds such as deferasirox [6]. Therefore, increasing the solubility of deferasirox has a great importance, to improve its oral bioavailability. In the case of Exjade^®^, the commercial preparation of deferasirox, this step is overcome by the formulation of a tablet for oral suspension in which sodium lauryl sulphate is used as a solubilizing agent to improve the dissolution of DFX.

Due to the higher dose required for DFX to exert its therapeutic benefits, re-formulation of DFX into a new oral dosage form that has higher solubility and bioavailability is still desired. The dosage reduction to diminish its side effects and improvement of patient compliance particularly for pediatrics is important. Therefore, to enhance its oral bioavailability, increasing its aqueous solubility is crucial. So far, few studies were performed to improve the solubility of DFX, such as encapsulated imidazole-modified DFX into polymeric micelles as a nano carrier [7], or to increase the solubility of DFX by decreasing its particle size and using sodium lauryl sulfate or Pluronic F127 as surfactants [8].

Lipid-based formulations (LBFs) are one of the efficient technologies to improve aqueous solubility, and thus to improve the bioavailability of lipophilic drug molecules [9]. Among the LBFs, self-nanoemulsifying drug delivery systems (SNEDDSs) have gained great attention, as an approach to improve oral bioavailability of drug substances which have low aqueous solubility. SNEDDSs are isotropic mixtures of the drug, oil, and hydrophilic surfactants and co-surfactant/co-solvent. Instantaneously, under dilution and mild agitation provided by the peristaltic motility in the gastrointestinal tract, they can form fine oil in water emulsion which has a globule size of less than 50 nm [10]. The oil component of the SNEDDS can be short chain triglycerides, medium chain triglycerides, or long chain triglycerides with different degrees of saturation, while surfactants are mainly non-ionic surfactants with a high hydrophilic lipophilic balance (HLB)value of more than 12 [11].

In addition, SNEDDSs are unique drug delivery systems that are characterized by thermodynamic stability of the nanoemulsion formed, rapid onset of action, ease of preparation process, and scale-up, in comparison with other lipid-based drug delivery systems [12], which make them attractive for industrial manufacturing.

Conventional liquid SNEDDSs are incorporated into a soft gelatin capsule; however, on long term storage, they could face some limitations, like precipitation at lower temperatures, drug leakages, excipient-capsule incompatibility, and handling and stability issues [13]. In order to overcome these limitations, combining the advantages of traditional SNEDDSs formulations and the solid dosage form by incorporating liquid SNEDDSs formulations into solid carrier and converting to solid SNEDDS (S-SNEDDSs) formulations by using different techniques, like spray drying or by adsorbing into porous carriers, result in free-flowing powder which can be formulated as powders, granules, pellets, and tablets, or filled into capsules [14].

To the extent of our knowledge, there has been no research study conducted to improve the solubility or oral bioavailability of DFX by developing a SNEDDS formulation. Thus, the aim of this research study is, for the first time, to develop and characterize a novel DFX-SNEDDS in order to increase its solubility and to potentially improve its oral bioavailability in order to evaluate the in vitro cytotoxicity effects of the optimized DFX-loaded SNEDDS formulation. Furthermore, the optimized DFX loaded SNEDDS would be incorporated into S-SNEDDS formulation, by adsorbing into different porous carriers to allow to compare their dissolution behavior with the commercially available tablet formulation of DFX.

## 2. Results and Discussions

### 2.1. Analytical Method for DFX Analysis

The calibration curves used to analyze the concentration of DFX in different excipients showed a good linear relationship over the concentration range of 2.5–12.5 μg/mL of DFX, with a high correlation coefficient (*R*^2^ = 0.9987) and precise intra- and inter-day variation (<2%) and accurate mean recovery(>98%). The limit of detection (LOD) and limit of quantification (LOQ) values were 0.596 and 1.806 μg/mL, respectively.

### 2.2. Optimization of DFX Loaded SNEDDS

#### 2.2.1. Equilibrium Solubility of DFX in the SNEDDSs Components

The development of a successful SNEDDS formulation depends on choosing the right excipients which reveal the best solubilizing potential for the drug, to ensure maximum drug loading and to keep the emulsification performance. In addition, the solubility of the drug in the proper excipients has a major role in the stability of the final formulation; if the solubility of the drug is not enough, then precipitation could happen in the early stages of development [15].

**Figure 1:**
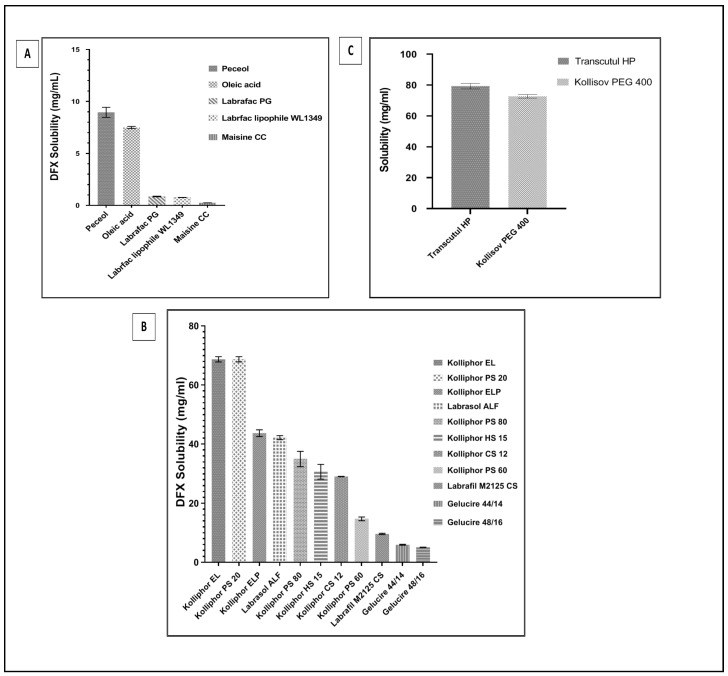
The solubility of deferasirox (DFX) in (**A**) different types of oils, (**B**) different types of surfactants, and (**C**) different types of co-surfactants. Each value represents the mean ± SD (*n* = 3).

Considering the therapeutic dose strength of DFX, a higher drug loading capacity of SNEDDS formulation is crucial. The initial choice of the type of oil, surfactant, and co-surfactant used in the compositions of SNEDDSs formulations were decided based on the maximum drug solubilizing capacity. As it is well-known, higher drug solubility in SNEDDS formulation components enables higher loading capacity in the SNEDDS [16]. The mean concentration of DFX saturated solubility in oil, surfactant, and co-surfactant screened are shown in Figure 1A–C, respectively.

The oil phase of SNEDDS formulation plays a major role in solubilizing the required doses of the drug and transporting it via the intestinal lymphatic system. Peceol (glycerol monooleate) was chosen as the oil phase which revealed the maximum DFX solubilization value of 8.95 ± 0.48 mg/mL (Figure 1). As reported in the literature, Peceol has been successfully used in many SNEDDS formulations because of its solubilizing capacity and ability to reduce P-glycoprotein (Pgp)-mediated efflux [17,18]. Kolliphor EL (glycerol polyethylene glycol ricinoleate), as the surfactant, displayed a solubility value of 68.71 ± 1.57 mg/mL. It is a hydrophilic non-ionic surfactant, which has a value of 12–14 HLB, has the ability to inhibit P-glycoprotein activity in the gut wall, thus increasing absorption after oral administration [19], and also inhibiting different Cytochrome enzymes, therefore, it increases oral bioavailability [20]. Transcutol HP (diethylene glycol monoethyl ether), as the co-surfactant has a quite high DFX solubility capability (79.26 ± 3.05 mg/mL), and it also has a capability to construct a stable interfacial film with surfactants due to its HLB value of 4.2. Transcutol HP has been previously reported to have a role in enhancing the bioavailability of poorly soluble drugs [21]; thus, it was concluded that Peceol, Kolliphor EL, and Transcutol HP are the promising components for developing SNEDDS of DFX, and they were checked for the formation of stable nano-size emulsion upon dispersion in water.

#### 2.2.2. Construction of Pseudo-Ternary Phase Diagrams

**Figure 2:**
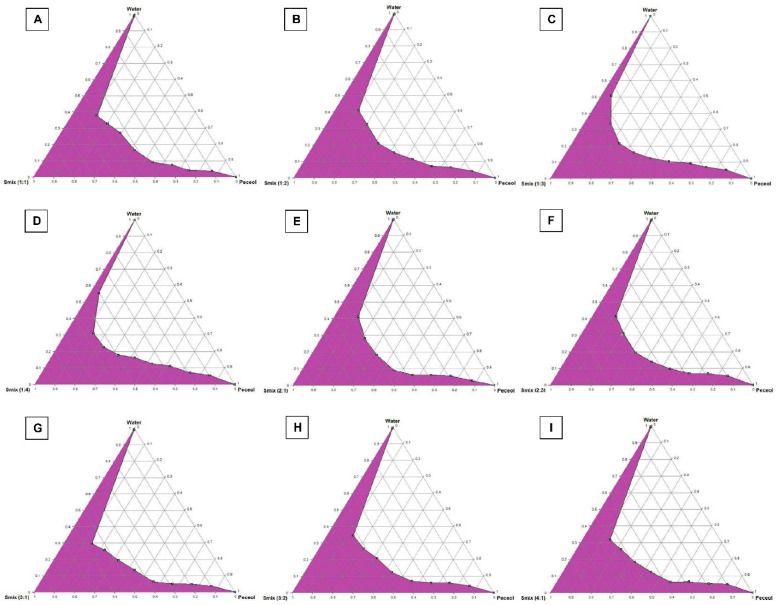
Pseudo-ternary phase diagrams of Peceol, Kolliphor EL, and Transcutol at Smix ratios (**A**) 1:1, (**B**) 1:2, (**C**) 1:3, (**D**) 1:4, (**E**) 2:1, (**F**) 2:3, (**G**) 3:1, (**H**) 3:2 and (**I**) 4:1. The colored region represents the nanoemulsion formation region.

The importance of pseudo-ternary phase diagram construction is to label the self-nanoemulsifying regions and to identify the appropriate concentrations of oil (Peceol), surfactant (Kolliphor EL) and co-surfactant (Transcutol HP) for the formulation of a stable SNEDDS formulation, which will not lose its solvent capacity of DFX and precipitate after dilution with the body fluids [22]. Nanoemulsion formation is defined as clear and homogenous systems obtained upon dilution; the area of nanoemulsion is represented by the colored region in the pseudo-ternary phase diagrams in Figure 2A–I.

The pseudo-ternary phase diagrams at different Smix (KolliphorEL: Transcutol HP) ratios (1:1, 1:2, 2:1, 2:3, 3:1, 3:2 and 4:1) show clear-transparent systems formed when the oil content was 10%, while the bi-phasic system formed from higher oil percentage indicates a higher percentage of Smix form fine clear nanoemulsion [23]. However, increasing the surfactant ratio (Kolliphor EL) in Smix ratios(1:1, 2:1, 3:1 and 4:1) led to an increase in the region of nanoemulsion formation, while an increase in co-surfactant (Transcutol HP) ratio in Smix ratios (1:1, 1:2, 1:3, 1:4) decreased the region of nanoemulsion formation. This could be due to the fact that the surfactant forms a layer around the oil globule and efficiently reduces surface tension, though co-surfactants have little effect on reducing the interfacial tension directly; therefore, increases in co-surfactant concentration will not be enough to reduce the surface tension and maintain thermodynamic stability of the formulated systems upon dilution [24].

#### 2.2.3. SNEDDS Formation Assessment

Visual observations revealed that seven mixtures containing 10% Peceol as the oil component were clear transparent dispersions upon dilution with purified water for 100 times. The seven mixtures dispersed into nanoemulsion dispersions have a droplet size of less than 50 nm and Polydispersity index (PDI) of less than 0.3 as shown in Table 1.

### 2.3. Equilibrium Solubility of DFX Solubility in Selected SNEDDS Formulations

**Figure 3:**
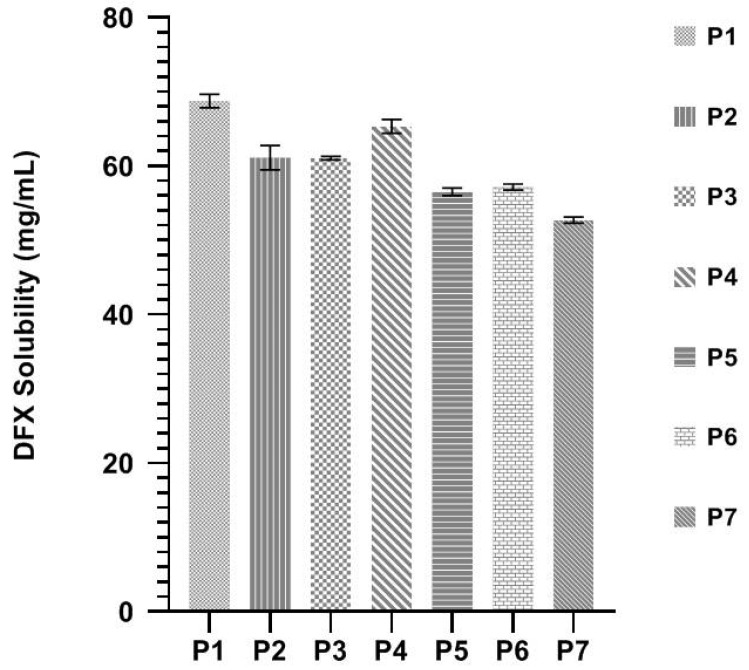
Solubility of DFX in selected SNEDDSs formulations. Each value represents the mean ± SD (*n* = 3).

Solubility results of DFX in the seven formulations are given in Figure 3, which shows that all SNEDDS formulations have DFX a solubility capability of more than 50 mg/mL. The surfactant and co-surfactant amount changes in SNEDDS formulations could not have a significant effect on the solubility of DFX.

### 2.4. Preparation of DFX Loaded SNEDDS Formulations

According to the equilibrium solubility results of SNEDDS formulations, each of the seven SNEDDS formulations loaded with 40, 45, or 50 mg of DFX were prepared for further investigation of the stability of DFX into the prepared SNEDDS formulations and droplet size measurement of the resulting nanoemulsions after dilution.

### 2.5. Characterization of DFX Loaded SNEDDS Formulations

#### 2.5.1. Droplet Size and PDI Determination

The droplet size of SNEDDS has a great impact on the rate and amount of drug which is dissolved and absorbed in the gastrointestinal tract after oral administration. Reduction in droplet size results in an increase in interfacial surface area, which leads to an improvement in absorption [25]. The PDI value of SNEDDS indicates the homogeneity of the droplet size distribution and a lower value of PDI indicates that the droplet size range distribution is highly homogenized.

Uploading 50 mg of DFX into the formulation resulted in higher droplet size and high PDI values, and DFX precipitated after 6 h out of the dispersion, which is a sign of instability of the nanoemulsions. As shown in Table 2, decreasing the amount of DFX uploaded and increasing the amount of surfactant, droplet size, and PDI values of SNEDDS were decreased, due to the ability of surfactant to form a layer around the emulsion droplet and reduce the interfacial energy, hence stabilizing the system against coalescence [26].

Among the evaluated formulations, two formulations P5-40 and P7-40 showed small droplet sizes of 14.72 ± 1.50, 15.77 ± 3.56 nm, and narrow PDI of 0.214 ± 0.036 and 0.174 ± 0.03, respectively. This fulfilled the requirements for SNEDDS, regarding their droplet size and PDI values; droplet size of less than 50 nm and low PDI values indicate a narrow size distribution of the emulsion formed. Therefore, P5-40 and P7-40-coded SNEDDS formulations of DFX were selected for further characterization studies.

#### 2.5.2. Thermodynamic Stability Studies

Kinetic stability of colloidal nano-sized carriers is investigated by subjecting formulations to different stress conditions; centrifugation cycle, heating-cooling cycle, and freeze-thaw cycle to differentiate nanoemulsion from emulsion formation, thus removing metastable formulations [27]. Both selected SNEDDS formulations of DFX, P5-40 and P7-40, showed no signs of precipitation, cracking, turbidity, or creaming following the application of centrifugation, heating-cooling cycles, and freeze-thaw cycles.

#### 2.5.3. Percentage Transmittance Determination (% T)

The nanoemulsion resulting from the dilution of SNEDDS formulations is an optically isotropic mixture of water, oil, and a mixture of surfactant and co-surfactant; hence, since the nanoemulsion formed is a single thermodynamically stable solution, the clarity of this solution should be determined. Both SNEDDS formulations P5-40 and P7-40 formed a clear dispersion with transmittance percentage values of 99.6% and 99.7%, respectively.

#### 2.5.4. Dispersibility Test Results

Visual observations showed that both selected SNEDDS formulations of DFX (P5-40 and P7-40) formed a clear nanoemulsion in less than one minute and this is referred to be grade A, according to the grading system mentioned in method Section 3.6.4. Grade A formulation indicates that the formulation is robust enough to self-emulsified in less than one minute to form a clear nanoemulsion, when exposed to dilution [28].

#### 2.5.5. Robustness to Dilution

Precipitation of a drug in vivo, when exposed to different dilutions in the pH ranges in the gastrointestinal tract, will affect the drug absorption and retard it [29]. Therefore, the robustness of dilution was checked by dilution P5-40 and P7-40 for 50 and 100 times with different dilutions which mimic the in vivo environment (pH 1.2, pH 4.5, pH 6.8, and pH 7.4).

As shown in Table 3, P5-40 and P7-40-coded SNEDDS formulations of DFX were stable at 50 and 100 times dilution at pH 1.2, 4.5, 6.8, and 7.4 and neither precipitation were formed, nor cloudiness or phase separation observed, even for 24 h, which indicates the stability of the reconstituted emulsion. The results confirmed that the formulations P5-40 and P7-40 were robust to dilution with different dilution volumes of various media, and the stability of DFX in the emulsion did not influence the dilution process.

#### 2.5.6. Effect of pH of the Dispersion Media on Droplet Size and PDI

Droplet size is an important parameter for the stability of nanoemulsion, precipitated drug. SNEDDS formulation one diluted in different pH ranges of gastrointestinal tract, ranging from acidic to basic, will lead to an increase in droplet size; therefore, droplet size should not change significantly upon changes in the pH of the GIT [30].

**Figure 4:**
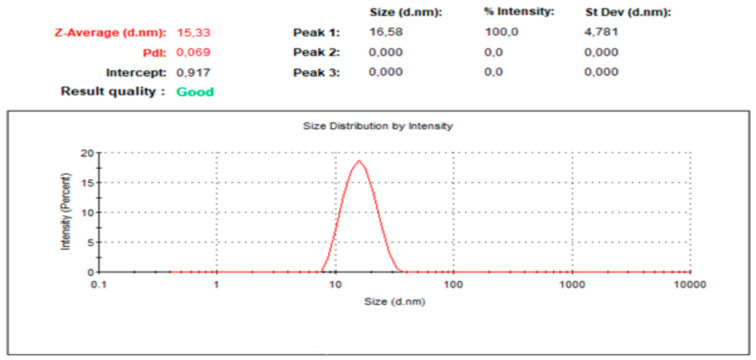
P5-40 formulation droplet size graphic reconstituted in distilled water.

As shown in Table 4, droplet size and PDI of the result and nanoemulsions did not influence changing the pH of the dispersion media used, an indication that these formulations have the ability to form a stable emulsion upon dilution in the gastrointestinal fluids. The droplet size distribution of formulation P5-40 in purified water is given in Figure 4, which shows one homogenized peak of 14.72–0.21 nm value and narrow size distribution.

#### 2.5.7. Transmission Electron Microscopy (TEM)

**Figure 5:**
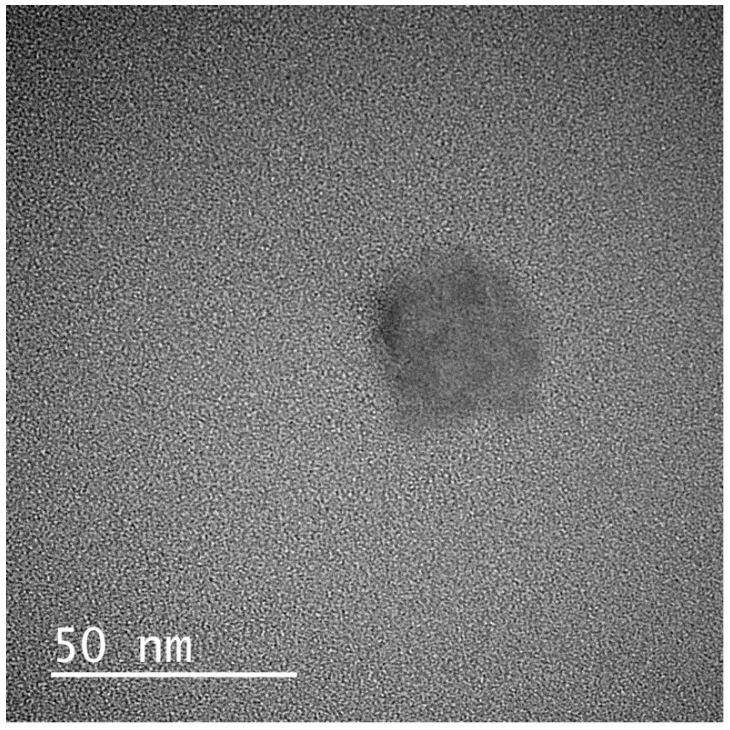
TEM image of the optimized SNEDDS formulation P5-40.

The nanoemulsion globules of the formulation P5-40 which was selected for further investigations were spherical with no signs of droplet aggregation, as shown in Figure 5.

### 2.6. In Vitro Cytotoxicity Studies

#### 2.6.1. MTT Assay

SNEDDS formulation P5-40, which has less surfactant concentration (67.5%) compared with SNEDDS formulation P7-40 (72%), was chosen for further in vitro cytotoxicity test. The cytotoxicity test revealed the potential of DFX and DFX loaded SNEDDS in inhibiting the growth of the test cell. The cytotoxic potential for SNEDDS formulation P5-40 was assessed in comparison with that of a negative control (cells without any treatment). In the research study, the human immortalized myelogenous leukemia cell line K562 was used and these cells were treated with 10, 30, and 40 μM of DFX consisting of the P5-40 formulation, placebo SNEDDS formulation (P5°), and pure DFX to examine if P5-40, P5°, and pure DFX have any effect on cell viability. The results indicated that cells that were exposed to P5-40, P5°, and pure DFX were comparable to cells without any treatment as a negative control.

**Figure 6:**
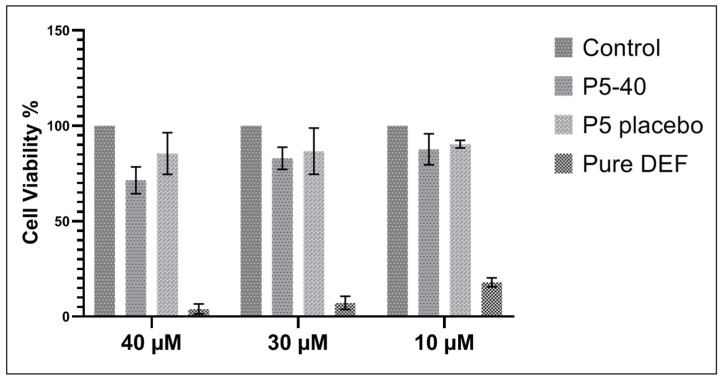
K562 cell viability of pure DFX, P5-40, and P5^0^ formulations at different concentrations of 10, 30, and 40 μM of DFX. (*n* = 3). (*p* value < 0.05).

The MTT data represented in Figure 6 illustrated that pure DFX showed low percent cell viability in a concentration-dependent manner compared with the negative control group and DFX loaded SNEDDS formulation (P5-40). The maximum cell death was produced at 40 μM of pure DFX where 3.99% cell viability was detected, while, at the same concentration, the cell viability percentage (%) of DFX loaded SNEDDS formulation (P5-40) was 71.44%, suggesting a least/non-toxic effect. This effect of the developed DFX loaded SNEDDS formulation is likely due to the formation of nanoemulsion upon dilution where DFX remained in the globule as an oil/water emulsion and resulted in less interaction with the cells [31].

#### 2.6.2. Investigating Cell Morphology and Cell Proliferation Using a Light Microscope

**Figure 7:**
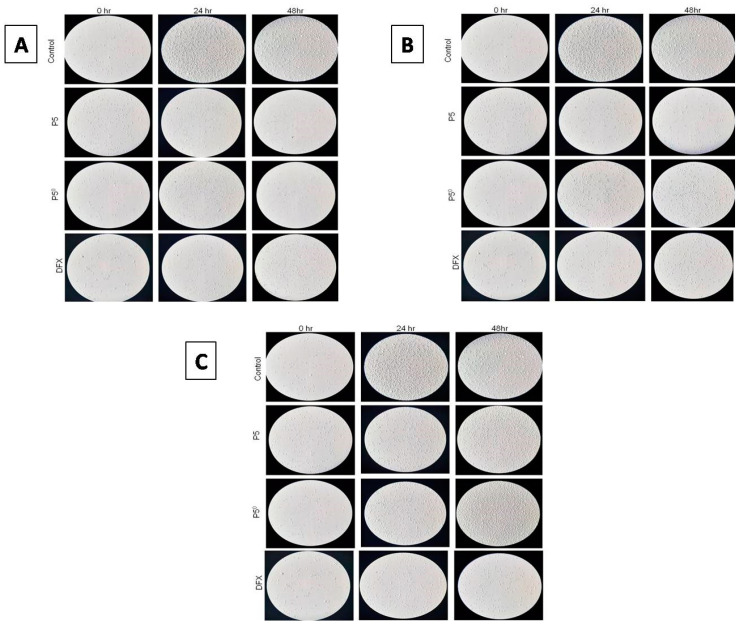
Light microscope images of K562 cells which were exposed to P5-40, P5^0^, and pure DFX of concentrations (**A**) 40 μM, (**B**) 30 μM, and (**C**) 10 μM for 24 and 48 h and control for comparison.

The images shown in Figure 7 reveal that both 30 and 40 μM of P5-40, P5^0^, and pure DFX had an antiproliferative effect on K562 cells, both at 24 and 48 h. The antiproliferative effect observed for the 30- and 40-μM concentrations have not been observed for the 10 μM of P5-40, P5^0^, and pure DFX at the same extend. Changes in the morphology of K562 cells were also observed at 30 and 40 μM of P5-40, P5^0^, and pure DFX at both 24 and 48 h. The circular shape of the K562 cells was disrupted at both time points, whilst the 40-μM concentration was affecting the morphology greatly. Moreover, as the cells were exposed to P5-40, P5^0^, and pure DFX more at 48 h, the morphological changes observed were considerable.

### 2.7. Development of DFX Loaded Solid-SNEDDS (S-SNEDDS)

Recently, adsorption to solid carriers has become the most intensively investigated approach to obtain S-SNEDDS formulations. Solid carrier employed in this research work (Neusilin UFL2, Neusilin US2, and Syloid XDP 3150) are recognized as safe (GRAS status) and could be effectively used as carriers to produce solid SNEDDS formulations [32]. Maximal oil adsorption was achieved based on the minimum amount of carrier material required for complete adsorption to obtain powder flow freely.

The oil adsorption capacity for the carriers was found to be equal for both Neusilin UFL2 and NeusilinUS2 (150 mg for 300 mg of SNEDDS formulation) and less in the case of Syloid XDP 130 (175 mg for 300 mg of SNEDDS formulation). The properties of the carriers are presented in Table 5; Syloid XDP 130 has the highest particle size and lower porosity compared with the other carriers that could lead to lower oil absorption capacity. According to adsorbing capacity, the P5-40 formulation solidified with three different adsorbents (NeusilinUS2, Neusilin UFL2, and Syloid XDP 130) which were prepared for further characterization. The components of each formulation (DFX-S-SNEDDS) are illustrated in Table 6.

### 2.8. Characterization of DFX-S-SNEDDS

#### 2.8.1. Fourier Transformed Infrared Spectroscopy (FT-IR)

The FT-IR spectrum for DFX as pure, DFX-S-SNEDDS formulations and carriers used are provided in the Supplementary Materials. FT-IR spectrum of pure DFX is shown in Figure S1. It revealed absorption bonds at 3317 cm^−1^ (OH, stretching), 1687 cm^−1^ (acid, conjugated C=O stretching), and 1584 cm^−1^ (aromatic, C=C stretching) [33]. The IR spectra for the DFX-S-SNEDDSs formulations (P5-40-UFL2, P5-40-US2, and P5-40-SYLOID) are presented in Figures S2–S4, respectively, while the IR spectra of Neusilin UFL2, Neusilin US2, and Syloid XDP 3150 are presented in Figures S5–S7, respectively. The data indicated that all the absorption bonds due to the functional group of DFX were presented in the three SNEDDS formulations (P5-40-UFL2, P5-40-US2, and P5-40-SYLOID), and there was no significant difference found in wave number (cm^−1^) in the spectrum of the prepared S-SNEDDS formulations. This finding indicates that there was no unwanted interaction between DFX and other used excipients in the study.

#### 2.8.2. *Scanning Electron Microscopy (SEM) Imaging*

**Figure 8:**
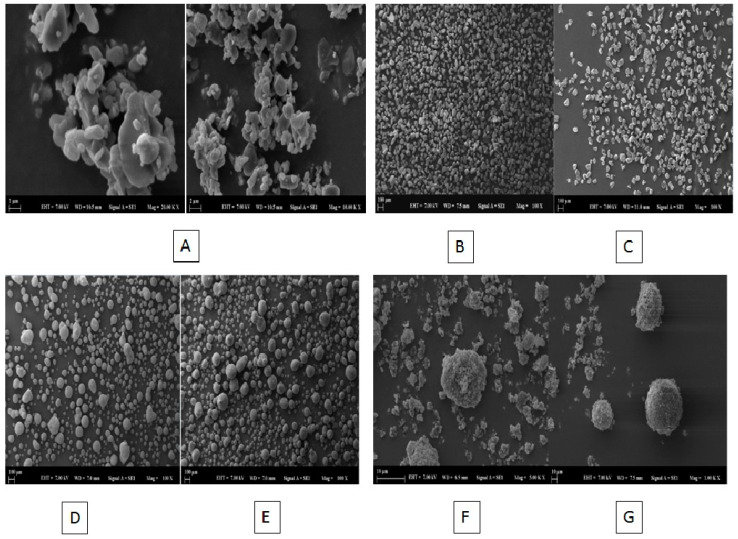
Scanning electron microscopy photographs of (**A**) Pure DFX, (**B**) Syloid XDP 3150, (**C**) P5-40-SYLOID, (**D**) Neusilin US2, (**E**) P5-40-US2, (**F**) Neusilin UFL2, and (**G**) P5-40-UFL2.

The SEM images of DFX, DFX-S-SNEDDs formulation, and carriers used for solidification are shown in Figure 8A–G. Figure 8A shows that pure DFX consists of asymmetrically shaped crystals as a mixture of small and large crystals. When comparing SEM images of S-SNEDDS formulations with the three types of carriers, Syloid XDP 3150 (Figure 8B) had irregular crystalline shape and the P5-40-SYLOID formulation kept the irregular shape of the Syloid XDP 3150 particles and had no oil globules (Figure 8C). In the case when both Neusilin^®^ UFL2 and Neusilin^®^ US2 were used as carriers, both carriers have a sphere-like shape (Figure 8D,F, respectively). Following mixing of L-SNEDDS with the solid carrier, the L-SNEDDS formulation was adsorbed totally into the internal Neusilin pores and both P5-40-US2 (Figure 8E) and P5-40-UFL2 (Figure 8G) kept the sphere-like shape of both carriers. Overall, these findings indicate the complete adsorption of liquid DFX-SNEDDS into the three types of carriers which were noticed by the absence of oil globules and disappearance of the DFX crystalline shape.

### 2.9. In Vitro Dissolution Studies of DFX-S-SNEDDS

**Figure 9:**
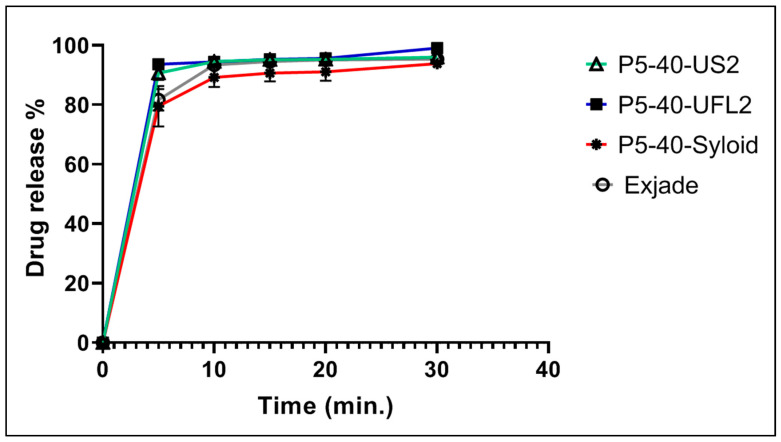
Drug release % of DFX from optimized S-SNEDDS formulated with different carriers and its commercial tablet (Exjade^®^) in phosphate buffer of pH 6.8 containing 0.5% Tween 20 (dissolution media recommended by the FDA). The data represent drug release (%) versus time (in min) in terms of mean ± SD (*n* = 3) (*p* < 0.05).

In vitro dissolution studies were conducted to evaluate the release characteristics of DFX from developed DFX-S-SNEDDS formulations (P5-40-UFL2, P5-40-US2, and P5-40-SYLOID) and to compare their drug release characteristics with that of the market product of DFX (Exjade^®^, Novartis, Switzerland) in a dissolution medium of phosphate buffer of pH 6.8 containing 0.5% Tween 20. The release profiles of DFX from S-SNEDDS formulations and Exjade^®^ are shown in Figure 9.

The dissolution data indicated that the release performance of DFX from S-SNEDDS formulations was significantly improved. Drug release occurred within 5 min. The Q5% observed by the P5-40-UFL2 formulation (93.6 ± 0.7%) and P5-40-US2 formulation (90.67 ± 2.35%) was significantly higher than that of the market tablet preparation of DFX (81.65 ± 2.10%) (*p* < 0.05). However, in the case of the P5-40-SYLOID formulation, the Q5% of DFX was found to be 82.95 ± 4.59%, which is quite closer to the concentrations of DFX released from the market product.

The increase in the percentage of DFX released from S-SNEDDS formulations is likely due to the spontaneous formation of nanoemulsion with small globules in the nano size once contacted with the dissolution media, which results in higher surface area, which permits a faster rate of drug release and extent [34]. That could induce a higher absorption and higher oral bioavailability of DFX. The enhancement of the dissolution profile of P5-40-UFL2 and P5-40-US2 could also be related to the lower particle size of both adsorbents compared with the particle size of the Syloid XDP 3150 used in the P5-40-SYLOID formulation, which offer a higher surface area for dissolution. In addition, Neusilin US2 and Neusilin UFL2 are highly porous solids that allow a quick entrance of dissolution medium into the pores and rapid emulsification [24].

When comparing the drug release within 5 min (Q5%) from P5-40-UFL2 and P5-40-US2, P5-40-UFL2 exhibited a higher Q5% value compared withP5-40-US2, which was aligned with Park’s study [35] reporting that Neusilin US2 used in the formulation of P5-40-US2 had a larger particle size and a similar specific surface area compared with Neusilin UFL2 which was used in formulation P5-40-UFL2. Therefore, this result could be due to the presence of larger numbers of long and narrow intra-particular pores where DFX could entrap inside these pores and cause a slightly less percentage of released DFX.

**Figure 10:**
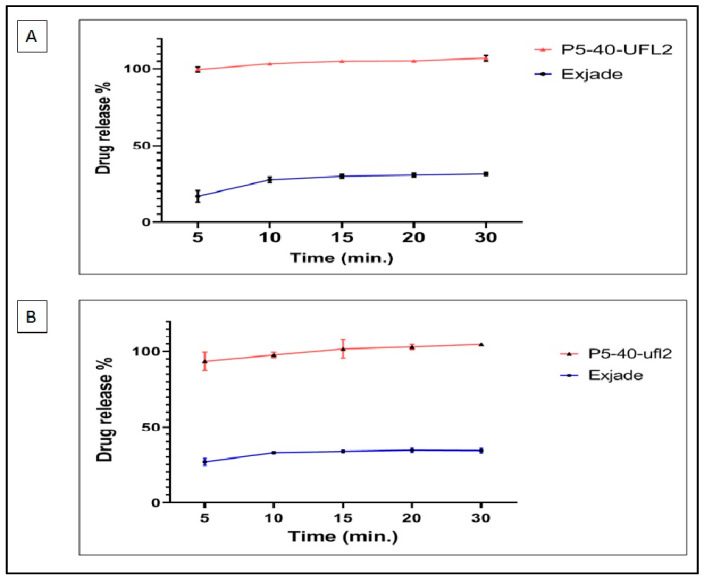
Drug release % of DFX from P5-40-UFL2 and its commercial tablet (Exjade^®^) in (**A**) phosphate buffer of pH 6.8 and (**B**) pH 1.2. The data represent drug release (%) versus time (in min) in terms of mean ± SD (*n* = 3) (*p* < 0.05).

P5-40-UFL2 exhibited the highest drug release within 5 min. P5-40-UFL2 was selected for performing a dissolution study to check DFX release in phosphate buffer of pH 6.8 without using Tween 20 and to compare with the DFX release from the market product (Exjade^®^). As illustrated in Figure 10A, 31.25% of DFX was released from the market product at 30 min, while for P5-40-UFL2, the percentage released at 5 min was 99%. DFX release from formulation P5-40-UFL2 and market product Exjade^®^ in a medium of pH 1.2 was also performed to mimic the stomach pH (Figure 10B). This profile shows that the percentage released of DFX from P5-40-UFL2 is almost 3 times higher than that of market product Exjade.

These results of in vitro release tests emphasized that the aim of formulating DFX into SNEDDS formulations was successfully achieved for increasing its solubility. It has been previously explained by Ameeduzzafar et al. that surfactants and co-surfactants in SNEDDS reduce interfacial tension and that SNEEDS adsorbed to the carrier improves dissolution with high surface area, high porosity, small droplet size, rapid emulsification without the need for a special environment to obtain sink condition. [36]. Our dissolution data also supported that phenomenon.

### 2.10. Kinetic Analysis of DFX Release Data

The best fitting kinetic model for the in vitro release of DFX from S-SNEDDS formulations and for the market product (Exjade^®^) can be calculated from the highest values of the obtained determination coefficients (*R*^2^). Table 7 showed that the r^2^ values for DFX release from the market product (Exjade^®^), P6-40-UFL2, P6-40-US2, and P6-40-Syloid were the highest for the Korsemeyer–Peppas model with values of 0.842, 0.788, 0.914, and 0.848, respectively. The n value indicates the characteristic of release mechanisms [37]. An n value higher than 1.0 which was calculated for all formulations implies that DFX release from S-SNEDDS formulations follows the Super case-II transport release mechanism, which refers to the erosion of the polymer chain and explains the initial burst release [38].

## 3. Materials and Methods

### 3.1. Materials

DFX was kindly gifted from Sanovel Drug Company, Turkey. Labrafac, Lipophile WL1349, Labrafac PG, Peceol, Transcutol HP, Labrasol ALF, Labrafil M2125, Maisine, Gelucire 44/14 and Gelucire 48/16 were kindly gifted from Gatteffosse (Lyon, France). Kolliphor PS20, Kolliphor PS60, Kolliphor PS80, Kolliphor CS12 and Kolliphor HS15, Kolliphor EL, Kolliphor ELP, and Kollisov PEG 400 were kindly gifted from BASF (Ludwigshafen, Germany). Syloid XDP 3150 was gifted from Grace (Columbia, Maryland, USA). Neusilin UFL2 and Neusilin US2 were gifted from Fuji chemical (Tokyo, Japan). All other chemicals were of analytical grade.

### 3.2. Analytical Method for DFX Analysis

The analysis of DFX was performed using UV-VIS spectrophotometer (UV-1601; Shimadzu, Kyoto, Japan) at 245 nm. The analysis method was validated according to International Conference on Harmonization (ICH) Q2. The diluent consisted of acetonitrile and methanol (50:50, *v*/*v*) [39]. It was also used for the analysis of the DFX solubility in different excipients and SNEDDSs formulations.

### 3.3. Optimization of Loaded L-SNEDDS

#### 3.3.1. Equilibrium Solubility of DFX in the L-SNEDDS Components

Equilibrium solubility of DFX in different types of oils, surfactants, and co-surfactants were determined using the shaking flask method. An excess amount of DFX was added to 1 mL of each component in an Eppendorf tube, solid excipients were heated by using a water bath at 45 °C whenever necessary to facilitate melting, stirred by using a vortex mixer (Nuve NM 110, Biobase^®^, Shandong, China). The sealed vials were kept on an orbital shaker (Model 420, Thermo Electron Corporation^®^, Waltham, MA, USA) at 37 ± 0.5 °C for 48 h to attain an equilibrium, and then centrifuged (Model D-7200, Hettich^®^, Tuttlingen, Germany) at 5000 rpm for 15 min. [36]. The supernatant was filtered through PTFE filter membrane (0.45 μm, Alwsci^®^, Hangzhou, China) and analyzed at UV Spectrophotometer, as described in Section 3.2.

#### 3.3.2. Construction of Pseudo-Ternary Phase Diagram

To understand the phase behavior and to observe the SNEDDS formation ratios of the SNEDDS excipients which are oil, surfactant, and co-surfactants, a pseudo-ternary diagram was constructed by using an aqueous titration technique [40], with oil, Smix (the mixture of surfactant to co-surfactant), and water, each representing an apex of the triangle. Based on solubility studies stated above, Peceol, Kolliphor EL, and Transcutol were selected as the oil phase, the surfactant, and the co-surfactant phase of SNEDDS, respectively.

Surfactant (Kolliphor EL) and co-surfactant (Transcutol) were mixed (Smix) with different weight ratios (1:1, 1:2, 1:3, 4:1, 2:1, 2:3, 3:1, 3:2, and 4:1) for each phase diagram. Specific Smix ratio was mixed with oil (Peceol) with different ratios (from 1:9 to 9:1) in separated glass vials stirred at 50 rpm. Then, they were titrated with purified water drop by drop and, vortexed after each addition at room temperature was observed by the naked eye for any turbidity or phase changes were reported and the weight of the water was recorded for use in the coming concentration measurements for constructing the phase diagram. The nine phase diagrams, one for each Smix, were constructed by using a ProSim Ternary Phase Diagram Software (Stratege, Cedex^®^, Toulouse, France).

#### 3.3.3. SNEDDS Formation Assessment

In order to check nanoemulsion formation, each oil and Smix ratio previously prepared for pseudo-ternary phase diagram preparation was evaluated for the formation of nanoemulsion by diluting 50 mg of each of the mixtures to 50 mL with purified water (Merck Milli Q, Darmstadt, Germany) in a volumetric flask, and checked visually for the formation of nanoemulsion. The mixtures which formed stable and transparent dispersions were subjected to droplet size measurement and PDI by using ZetaSizer Nano ZS (Malvern 1000 HS^®^, Worcestershire, UK) [41]. These measurements were done at 37 °C after an equilibration time of 120 s. Each sample was measured 3 times with 12–17 runs for each measurement.

### 3.4. Equilibrium Solubility of DFX in Selected SNEDDSs Formulations

The goal of a SNEDDS formulation is to develop a formulation that is capable to upload maximum amount of DFX into 1 mL of the SNEDDS mixture. Therefore, equilibrium solubility of DFX was measured in the selected SNEDDSs formulations, by applying the same procedure for measuring DFX solubility in the excipients. An excess amount of DFX was added to 1 mL of each of the combinations in an Eppendorf tube, stirred by using a vortex mixer (Nuve NM 110, Biobase^®^, Shandong, China). The sealed vials were kept on the orbital shaker (Model 420, Thermo Electron Corporation^®^, Waltham, MA, USA) at 37 ± 0.5 °C for 48 h to attain an equilibrium, then centrifuged (Model D-7200, Hettich^®^, Tuttlingen, Germany) at 5000 rpm for 15 min. The supernatant was filtered through a PTFE filter membrane (0.45 μm, Alwsci^®^, Hangzhou, China) and analyzed at a UV Spectrophotometer, as described in Section 3.2.

### 3.5. Preparation of DFX Loaded SNEDDS Formulations

Based on solubility data for DFX in the selected formulations, DFX loaded SNEDDS (DEF-SNEDDS), which differ by amount loaded, were prepared by adding accurately weighted DFX to 1 mL of each of selected formulations and, mixing using a magnetic stirrer at 50 rpm for 30 min to allow solubilization. The mixtures were kept at room temperature in tightly closed glass bottles for further use. Table 8 below illustrates SNEDDS formulation codes and the composition of each formulation.

### 3.6. Characterization of DFX Loaded SNEDDS Formulations

#### 3.6.1. Droplet Size and PDI Determination

In order to measure the droplet size and PDI of SNEDDS formulations, SNEDDS formulations 100-fold diluted in purified water were prepared and analyzed through a ZetaSizer (Malvern 1000 HS, Worcestershire, UK). These measurements were done at 37 °C after an equilibration time of 120 s, and each sample was measured 3 times with 12–17 runs for each measurement. The formulations which had a droplet size of less than 50 nm and optimum PDI values closer to zero were subjected to further characterization tests [41].

#### 3.6.2. Thermodynamic Stability Studies

Thermodynamic stability studies were performed to evaluate visually the phase separation and effect of temperature variations on DFX loaded SNEDDS stability comprising centrifugation, heating–cooling cycle and freeze–thaw cycle [15].

The selected DFX loaded SNEDDS formulations which passed the requirements for droplet size and PDI were diluted with purified water (1:20) and centrifuged at 3500 rpm for 30 min to find out their stability as an isotropic single-phase system. Formulations that showed no signs of phase separation, creaming, or cracking were subjected to three heating cycles and three cooling cycles in which samples were incubated at 4 and 45 °C for 48 h. The formulations which passed heating and cooling cycles were more subjected to three freeze–thaw cycles at temperatures between −20 °C and 25 °C in a deep freezer for 48 h minimum [42]. Formulations which passed the stability test were subjected to further characterization.

#### 3.6.3. Percentage Transmittance Determination (% T)

Nanoemulsions obtained from 100-fold dilution of DFX loaded SNEDDS in purified water were checked for their turbidity by measuring the percent transmittance (T, %) using UV–visible spectrophotometer at 638 nm; a blank used was purified water [43].

#### 3.6.4. Dispersibility Test

The efficiency of self-emulsification of DFX loaded SNEDDSs formulations were assessed by using a standard USP-dissolution apparatus-II. To be specific, 1mL of each formulation was added separately to 500 mL distilled water pre-heated at 37 ± 0.5 °C with continuous gentle agitation at 50 rpm [44].

The time and efficacy for self-emulsifying were evaluated according to the following grading system: Grade A: refers to rapidly forming (within 1 min) nanoemulsion, having a clear or bluish appearance; Grade B: indicated that emulsion rapidly forming (within 2 min), slightly less clear nanoemulsion, having a bluish white appearance; Grade C: being fine milky emulsion that was formed within 2 min; Grade D: was a dull-greyish white emulsion having slightly oil appearance that was slow to emulsify (longer than 2 min); Grade E: represented a formulation, exhibiting either poor or minimal emulsification with large oil globules present on the surface (longer than 3 min) [42].

#### 3.6.5. Robustness to Dilution

In order to simulate in vivo environmental conditions and to examine the effects of dilution in solutions which mimic pH of physiological fluids of the gastrointestinal system, 50 and 100 times dilution of DFX loaded SNEDDS formulations into 0.1N HCl and phosphate buffer of different pH values of 4.5, 6.8, and 7.4 were performed. The prepared nanoemulsions were stored at ambient conditions for 12 h and checked visually for any phase separation or precipitation formation [45].

#### 3.6.6. Effect of pH of the Dispersion Media on Droplet Size and PDI

The stability of DFX loaded SNEDDS formulations in different pH buffer solutions (pH 1.2, Ph4.5, pH 6.8 and, pH 7.4) was checked by 100-fold dilution in each of the buffer solutions, then subjected to droplet size and PDI measurements by ZetaSizer (Malvern 1000 HS, Worcestershire^®^, UK), and the values were compared with droplet size and PDI that resulted from dispersions in purified water [46].

#### 3.6.7. Transmission Electron Microscopy (TEM)

For observing the surface morphology, SNEDD formulations were diluted 1:100 with purified water and the prepared samples were dropped onto the TEM grid (carbon coated, 300 mesh copper grid). The excess volume of the sample was removed with a filter paper. Samples were loaded into the microscope after 5 s of plasma cleaning. The images were captured and analyzed by a Digital Micrograph with JEM-ARM200, 200 kV, JEOL (Tokyo, Japan) [36].

### 3.7. In Vitro Cytotoxicity Studies

To evaluate the relative safety of the selected DFX loaded SNED formulation, P5-40 coded SNEDDS formulation was selected for in vitro cytotoxicity study which had less concentration of surfactant to avoid unexpected irritation of gastrointestinal tract [47]. The cytotoxic effects of DFX loaded SNEDDS formulation (P5-40) and the same drug-free SNEDDS formulation (P5°) were compared to that of pure DFX itself (Pure DFX). For this purpose, the human immortalized myelogenous leukemia cell line, K562, was used. K562 is a previously established and well-characterized leukemia cell line from American Type Culture Collection (Rockville, MD, USA). In recent studies, K562 cells are highlighted and used promising venues for various diseases as well as cancer research. These cells were maintained in RPMI-1640 medium (Biological Industries, Bait HaEme, Israel) supplemented with 10% fetal bovine serum (FBS, Sigma-Aldrich), penicillin (64 μg/mL), streptomycin (0.1 mg/mL) and L-glutamine at 5% CO_2_ and at 37 °C. Cell survival was assessed using an MTT test.

#### 3.7.1. MTT Assay

To quantify cell viability and proliferation of K562 cells after treatment with selected DFX loaded SNEDDS (P5-40), MTT assay was applied by using Cell proliferation Kit I (MTT) (Roche, Penzberg, Germany); 100 μL of K562 cells at a concentration of 4 × 10^4^ cells/mL in culture medium were seeded into each well of a flat-bottomed 96- well plate and incubated 24 h in 5% CO_2_ and at 37 °C. Thereafter, these cells were treated with 10, 30, and 40 μM of DFX loaded SNEDDS formulation (P5-40), placebo SNEDDS (P5°), and pure DFX and incubated for 24 h in 5% CO_2_ and at 37 °C. Following this, 10 μL of MTT solution was added into each well, mixed gently, and incubated at 37 °C for 4 h. Then, formazan crystals were dissolved by adding 100 μL DMSO into each well [31]. The absorbance was read at 570 nm using a microplate reader (VersaMax™, Molecular Devices LLC, San Jose, California, USA). Untreated cells were considered as experimental control in line with the literature and the MTT protocol and a similar protocol was applied in triplicate. The absorbance measurement for the research study was performed at 630 nm, where neither MTT nor formazan absorbs, to eliminate possible errors. The cytotoxic activity was expressed as cell viability (%), the concentration of viable cells, and all experiments were performed in triplicate.

(1) $$\mathrm{Cell}\;\mathrm{viability}\;(\%)=\frac{\left(Absorbance\;of\;treated\;cells-Absorbance\;of\;blank\right)}{\left(Absorbance\;of\;control-Absorbance\;of\;blank\right)}\times 100$$

#### 3.7.2. Investigating Cell Morphology and Cell Proliferation Using Light Microscope

The morphology and proliferation of K562 cells were investigated under a light microscope (Leica Microsystems, Wetzlar, Germany). Cells were incubated for 24 and 48 hat 10, 30 and 40 μM of DFX loaded SNEDDS formulation (P5-40), placebo SNEDDS (P5°), and pure DFX, in order to see the cytotoxic effect to them.

### 3.8. Development of DFX Loaded Solid-SNEDDS (S-SNEDDS)

The solid DFX loaded SNEDDS formulations were attained by adsorbing DFX-SNEDDS formulation (P5-40) on solid carriers. Three different solid carriers were used: Neusilin^®^, US2, Neusilin^®^ UFL2 (Fuji Chemical, Tokyo, Japan), and Syloid^®^ XDP 3150 (Grace, Columbia, Maryland, USA) for preparing different batches of DFX-S-SNEDDS formulations. The oil adsorption capacity was measured for each solid carrier, which is defined as the amount of porous carrier required for transforming the unit dose of liquid oily formulation into the solid free flowing powder [48]. The 200 mg of each carrier was placed separately in a mortar and the formulation P5-40 was added drop wisely with good mixing after each addition until a non-sticky free flowing powder was obtained and the weight of P5-40 formulation used was recorded [49].

### 3.9. Characterization of DFX-S-SNEDDS

#### 3.9.1. Fourier Transformed Infrared Spectroscopy (FT-IR)

FT-IR analysis of DFX, prepared DFX-S-SNEDDS and pure carriers (Neusilin^®^ UFL2, Neusilin^®^ US2 and Syloid^®^XDB 3150) were carried out. The spectra were recorded using Fourier transform infrared spectrophotometer (Perkin Elmer Spectrum One, MA, USA) in the range of 4000–650 cm^−1^. The background was taken with air before shooting. By applying pressure to the sample, the peaks are provided more clearly [50].

#### 3.9.2. SEM Imaging

Scanning electron micrographs for DFX, DFX-S-SNEDDSs formulation, and pure carriers (Neusilin^®^ UFL2, Neusilin^®^ US2, and Syloid^®^ XDB 3150) were taken. Powder samples were woven on double-sided carbon bands and excess powders were swept with nitrogen gases. Carbon bands containing sample powders placed on the sample holders were subjected to the Gold–Palladium alloy coating process for 75 s using the Quorum SC7620 sputter coated (UK). Images with Zeiss Evo LS 10 scanning electron microscope (Carl Zeiss Microscopy GmbH, Jena, Germany) have been implemented. The images were obtained under a 7 kV acceleration voltage using the secondary electron detector [43].

### 3.10. In Vitro Dissolution Studies of DFX-S-SNEDDS

In vitro dissolution studies were performed for optimum DFX loaded S-SNEDDS formulation (P5-40) solidified with the different adsorbents mentioned above and a market product of DFX (Exjade^®^, Novartis, Switzerland), using a USP dissolution apparatus II (Sotax Smart AT7, Thun, Switzerland). As specified by the FDA [51], the paddle rotation speed was 75 rpm maintained at 37 ± 0.5 °C in 900 mL phosphate buffer with a pH value of 6.8 containing 0.5% Tween 20 as dissolution medium. A 3-mL volume of the aliquots was withdrawn at predetermined time points (5, 10, 15, 20, and 30 min), and filtered through a PTFE filter membrane (0.45 μm, Alwsci^®^, Hangzhou, China). The S-SNEDDS formulation (weight differ according to different carriers) which corresponded to 40 mg of DFX was added to each of the vessels. The blank used for S-SNEDDS formulation was S-SNEDDS formulation without DFX to avoid interferences from the excipients used in the formulation [52], while for the market product, the blank used was the same dissolution medium. These experiments were carried out in triplicate for each formulation.

Dissolution studies were performed in phosphate buffer at pH 6.8 without surfactants addition to see the effect of formulating DFX into SNEDDS formulation where the optimum formulation was compared with the market product. The other dissolution parameters were kept constant, except the dissolution medium of phosphate buffer with a pH value of 6.8 ± 0.05. Besides, the release of DFX from P5-40-UFL2 and market product was performed in a dissolution medium of pH 1.2 ± 0.05 while at the same time keeping the other dissolution parameter constants to mimic gastrointestinal media.

The amount of DFX dissolved in each medium was determined by separated validated spectrophotometric analysis method (UV-1601; Shimadzu, Japan) at 245 nm after a proper dilution with the medium used.

The amount of drug released into the medium was calculated (Equation (2)) and accordingly, the percentage of drug released was calculated according to Equation (3). The drug released (%) was plotted against time points.

(2) $$\mathrm{Amount}\;\mathrm{of}\;\mathrm{drug}\;\mathrm{released}=\frac{\mathrm{Concentration}\times \mathrm{Dissolution}\;\mathrm{path}\;\mathrm{volume}\times \mathrm{Dilution}\;\mathrm{factor}\;}{1000}$$

(3) $$\mathrm{Drug}\;\mathrm{release}\;\left(\%\right)=\frac{\left(\mathrm{Amount}\;\mathrm{of}\;\mathrm{drug}\;\mathrm{released}\;\mathrm{into}\;\mathrm{medium}\right)}{\mathrm{Total}\;\mathrm{amount}\;\mathrm{of}\;\mathrm{drug}}\times 100$$

### 3.11. Kinetic Analysis of DFX Release Data

For analyzing the in vitro release data, the in vitro release profile for market product and DFX S-SNEDDS of different carriers were fitted in various kinetic models. Models Zero order, First order, Higuchi model, Hixson–Crowell and Korsemeyer–Peppaswerewere applied, analyzed, and determination coefficients (*R*^2^) were calculated for each model.

The drug release rate in the Zero order model is independent of its concentration in the systems (Equation (4)), while for the First order model, the release rate is concentration dependent (Equation (5)). Higuchi model describes the release of drugs from the insoluble matrix as a square root of time-dependent process based on Fickian diffusion (Equation (6)). The Hixson–Crowell model describes the drug release from systems where surface area and diameter of particles or tablets changing with time (Equation (7)). The Korsemeyer–Peppas model describes drug release from a polymeric system (Equation (8)) [37].

(4) Qt=Q0+k0t

*K*_0_ is zero-order rate constant expressed in units of concentration/time and t is the time.

(5) Log Ct=LogC0−kt/2.303

*C*_0_ is the initial concentration of drug and K is first order constant.

(6) $$Qt=Kt_{1/2}$$

*K* is the constant reflecting the design variables of the system.

(7) $$Q0^{1/3}-Qt^{\frac{1}{3}}=K_{HC}t$$

*Q**_t_*** is the amount of drug released in time *t*, *Q*_0_ is the initial amount of the drug in tablet and *K_HC_* t is the rate constant for the Hixson–Crowell rate equation.

(8) $$\frac{M_{t}}{M_{\infty }}=Ktn$$

*M**_t_***/*M***_∞_** is fraction of drug released at time *t*, *k* is the rate constant and *n* is the release exponent.

### 3.12. Statistical Analysis

The results were compared by using two-way ANOVA test using GraphPad Prism 8.0.1 software. A *p* value of less than 0.05 was considered to be significant.

## 4. Conclusions

In the research study, a novel SNEDDS of DFX was developed consisting of Peceol (10%), Kolliphor EL (67.5%), and Transcutol HP (22.5%) as an oil phase, a surfactant, and a co-surfactant, respectively. The optimum SNEDDS formulation was further characterized, and it provided good thermodynamic stability with good self-emulsification efficiency. The developed clear nanoemulsion with a droplet size of 14.72 ± 1.50 nm upon dispersion with water was stable, and even stable against dilution and pH changes. The cell viability effect of SNEDDs formulation of DFX optimized in the research study was found to be relatively safe compared with the pure drug itself. Furthermore, the selected SNEDDS formulation of DFX was converted to DFX-S-SNEDDSs formulations by adsorbing it on different carriers (Neusilin UFL2, Neusilin US2, and Syloid XPD 3150). S-SNEDDSs formulations of DFX preserved the self-emulsification performance of the SNEDDS solidified with Neusilin UFL2 and exhibited the fastest in vitro DFX dissolution rate than those of the other adsorbents used, and even with its commercial product (Exjade^®^) in different dissolution media. These findings indicate enhanced dissolution of DFX by S-SNEED formulations.

Overall, our data support the solubility enhancement capability of DFX by optimized S-SNEDDS formulation, and furthermore, indicated that the optimized S-SNEDDS formulation of DFX has a potential for improving its oral bioavailability. Therefore, further in vivo studies would be investigated to confirm the bioavailability enhancement of the new solid SNEDDS formulation of DFX.

## Figures and Tables

**Table 1:** Droplet size and Polydispersity index (PDI) values of self-nanoemulsifying drug delivery system (SNEDDS) combinations for emulsification efficient (*n* = 3).

SNEDDS Formulation Code	Oil:Smix	Peceol:KolliphorEL:Transcutol HP (*w/w*, %)	Mean Droplet Size (nm) (±SD)	Mean PDI (±SD)
P1	1:9	10:45:45	20.46 ± 0.20	0.17 ± 0.04
P2	1:9	10:30:60	24.57 ± 0.14	0.19 ± 0.01
P3	1:9	10:60:30	16.04 ± 0.55	0.04 ± 0.01
P4	1:9	10:36:54	21.01 ± 0.09	0.17 ± 0.02
P5	1:9	10:67.5:22.5	15.30 ± 0.29	0.14 ± 0.01
P6	1:9	10:54:36	16.71 ± 0.89	0.19 ± 0.01
P7	1:9	10:72:18	15.13 ± 0.12	0.1 ± 0.01

**Table 2:** Effect of amount of DFX loaded into the SNEDDS formulations on the droplet size and PDI of SNNEDS (*n* = 3).

SNEDDS Formulation Code	DFX Amount (mg/mL)	Peceol:KolliphorEL:Transcutol HP (*w/w*, %)	Mean Droplet Size (nm) (±SD)	Mean PDI (±SD)
P1-50	50	10:45:45	128 ± 26.20	0.379 ± 0.08
P1-45	45		109.1 ± 1.70	0.498 ± 0.09
P1-40	40		73.71 ± 4.59	0.496 ± 0.01
P2-50	50	10:30:60	670 ± 147.95	0.885 ± 0.16
P2-45	45		239.4 ± 47.87	0.706 ±0.27
P2-40	40		189.5 ± 2.20	0.496 ± 0.01
P3-50	50	10:60:30	95.12 ± 14.70	1.000 ± 0.001
P3-45	45		120.5 ± 0.32	0.444 ± 0.01
P3-40	40		75.53 ± 63.4	0.546 ± 0.20
P4-50	50	10:36:54	165.6±2.20	0.352 ± 0.12
P4-45	45		140.62±74.18	0.686 ± 0.11
P4-40	40		122.3±1.82	0.348 ± 0.04
P5-50	50	10:67.5:2.5	39.20 ± 12.34	0.745 ± 0.28
P5-45	45		27.82 ± 0.83	0.803 ± 0.03
P5-40	40		14.72 ± 1.50 *	0.214 ± 0.036
P6-50	50	10:54:36	81.56 ± 2.12	0.544 ± 0.004
P6-45	45		41.28 ± 0.90	1.000 ± 0.001
P6-40	40		19.57 ± 0.30	0.578 ± 0.02
P7-50	50	10:72:18	33.79 ± 26.68	0.486 ± 0.12
P7-45	45		29.02 ± 12.44	0.478 ± 0.19
P7-40	40		15.77 ± 3.56 *	0.174 ± 0.03

* Values in bold represent the optimum droplet size values for formulations P5-40 and P7-40.

**Table 3:** Effect of dilution and pH on the stability of optimized DFX loaded SNEDD formulations.

SNEDD Formulation Code	0.1 N HCl pH 1.2		Phosphate Buffer pH 4.5		Phosphate Buffer pH 6.8		Phosphate Buffer pH 7.4	
	50 d.f	100 d.f	50 d.f	100 d.f	50 d.f	100 d.f	50 d.f	100 d.f
P5-40	Pass	Pass	Pass	Pass	Pass	Pass	Pass	Pass
P7-40	Pass	Pass	Pass	Pass	Pass	Pass	Pass	Pass

**Table 4:** Effect of pH of the dispersion media on droplet size (nm) and PDI (*n* = 3).

Formulation	Droplet Size (nm) and PDI				
	Purified Water	pH 1.2 (1 N HCl)	pH 4.5 (Phosphate Buffer)	pH 6.8 (Phosphate Buffer)	pH 7.4 (Phosphate Buffer)
P5-40	14.72–0.21	27.93–0.25	27.39–0.17	12.37–0.17	15.11–0.07
P7-40	15.77–0.17	28.91–0.24	33.98–0.35	12.56–0.16	14.97–0.06

**Table 5:** Oil adsorption capacity of porous carriers studied for the preparation of DFX- Solid-SNEDDS (S-SNEDDS).

Type of Carrier	Chemical Name	Appearance	Average Particle Size(μm) ^a^	Oil Adsorbing Capacity (mg)	SNEDDS to Adsorbent Ratio
Neusilin US2	Magnesium aluminometasilicate	White granules	106	150	2:1
Neusilin UFL2	Magnesium aluminometasilicate	Amorphous white powder	3.1	150	2:1
Syloid XDP 3150	Mesoporous silica	White free flowing powder	150	175	1.75:1

^a^ Data published by Fuji Chemical and Grace Company.

**Table 6:** Components of DFX-S-SNEDD formulations prepared for further characterization.

DFX-S-SNEDD ^a^ Formulation Code	Peceol:KolliphorEL:Transcutol HP (%, *w/w*)	Adsorbent Type
P5-40-US2	10:67.5:22.5	Neusilin U2 ^b^
P5-40-UFL2	10:67.5:22.5	Neusilin UFL2 ^b^
P5-40-SYLOID	10:67.5:22.5	Syloid XDP 3150 ^c^

^a^ 40 mg/mL of DFX was loaded into formulations, ^b^ 200 mg of weight adsorbent is required for 1 *g* of SNEDDS, ^c^ 175 mg of weight adsorbent is required for 1 g of SNEDDS.

**Table 7:** The determination of coefficient (*R*^2^) and release exponent (*n*) values for in vitro release profiles of the market product (Exjade^®^) and DFX S-SNEDD formulations prepared by different carriers.

Formulation	Zero Order	First Order	Higuchi Model	Hixon Crowell Model	Korsemeyer–Peppas Model	
	*R*^2^ *	*R*^2^ *	*R*^2^ *	*R*^2^ *	*R*^2^ *	*n*Value
Market product (Exjade^®^)	0.459	0.657	0.762	0.531	0.842 **	1.323
P6-40-US2	0.403	0.562	0.707	0.672	0.788 **	1.318
P6-40-UFL2	0.65	0.881	0.859	0.642	0.914 **	1.385
P6-40-Syloid	0.479	0.725	0.775	0.847	0.848 **	1.31

* *R*^2^ (R squared) value is a statistical measure of how close the data to the fitted regression line for each model. ** Values in bold represent the highest r2 value for each formulation in comparison with different models.

**Table 8:** Composition of the formulations prepared for further investigation.

SNEDDS Formulation Code	DFX Amount (mg/mL)	Peceol (*w*/*w*,%)	Kolliphor EL (*w/w*,%)	Transcutol HP (*w/w*,%)
P1-50			45	45
P2-50			30	60
P3-50			60	30
P4-50	50	10	36	54
P5-50			67.5	22.5
P6-50			54	36
P7-50			72	18
P1-45			45	45
P2-45			30	60
P3-45			60	30
P4-45	45	10	36	54
P5-45			67.5	225
P6-45			54	36
P7-45			72	18
P1-40			45	45
P2-40			30	60
P3-40			60	30
P4-40	40	10	36	54
P5-40			67.5	22.5
P6-40			54	36
P7-40			72	18

## Supplementary Materials

The following are available online at https://www.mdpi.com/1424-8247/13/8/162/s1, Figure S1. FT-IR Spectrum of Pure Deferasirox, Figure S2. FT-IR Spectrum of P5-40-Ufl2 Formulation, Figure S3. FT-IR Spectrum of P5-50-Us2 Formulation, Figure S4. FT-IR Spectrum of P5-40-Syloid, Figure S5. FT-IR Spectrum of Neusilin Ufl2 Carrier, Figure S6. FT-IR Spectrum of Neusilin US2 Carrier, Figure S7. FT-IR Spectrum of Syloid XDP 3150 Carrier.
