# Supplementary material for: A Solid Ultra Fine Self-Nanoemulsifying Drug Delivery System (S-SNEDDS) of Deferasirox for Improved Solubility: Optimization, Characterization, and In Vitro Cytotoxicity Studies
Source: Pharmaceuticals (Basel). 2020 Jul 24;13(8):162. doi: 10.3390/ph13080162 (PMC7465256; doi:10.3390/ph13080162)
Supplement: Supplementary file 1 [file pharmaceuticals-13-00162-s001.pdf]

A Solid Ultra Fine Self-nanoemulsifying Drug Delivery System(S-SNEDDS) of Deferasirox for Improved Solubility: Optimization, Characterization and *In vitro* Cytotoxicity Studies

Alaa Alghananim[1,2], Yıldız Özalp[1], Burcu Mesut[3], Nedime Serakinci[4,5], Yıldız Özsoy[3] and Sevgi Güngör[3]*

Supplementary material: Ft-ir spectra of pure deferasirox, p5-40-ufl2 formulation, p5-40-us2 formulation, p5-40-syloid formulation, neusilin ufl2 carrier, neusilin us2 carrier and syloid xdp 3150 carrier.

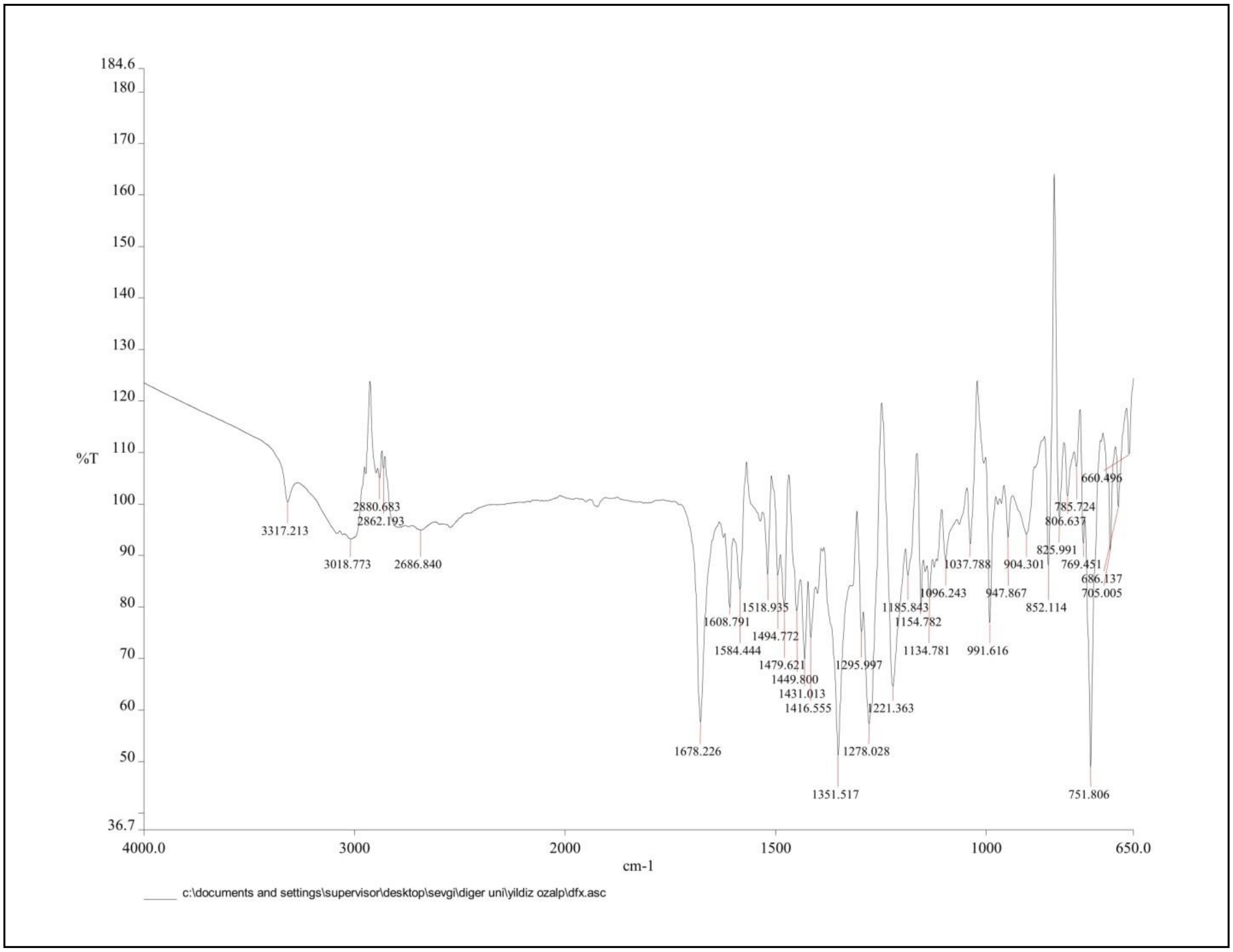

Figure S1. FT-IR spectrum of pure deferasirox.

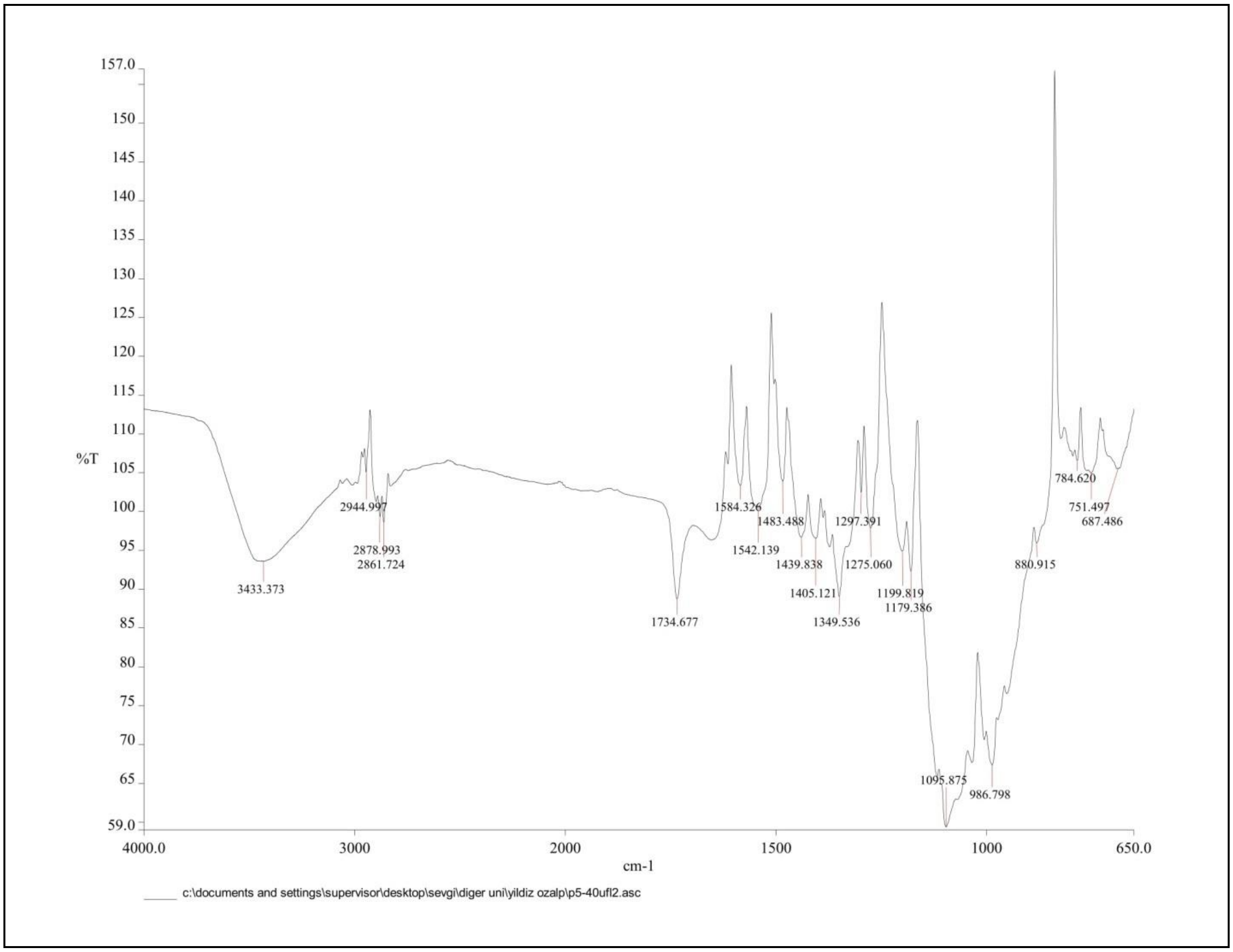

Figure S2. FT-IR spectrum of p5-40-ufl2 formulation.

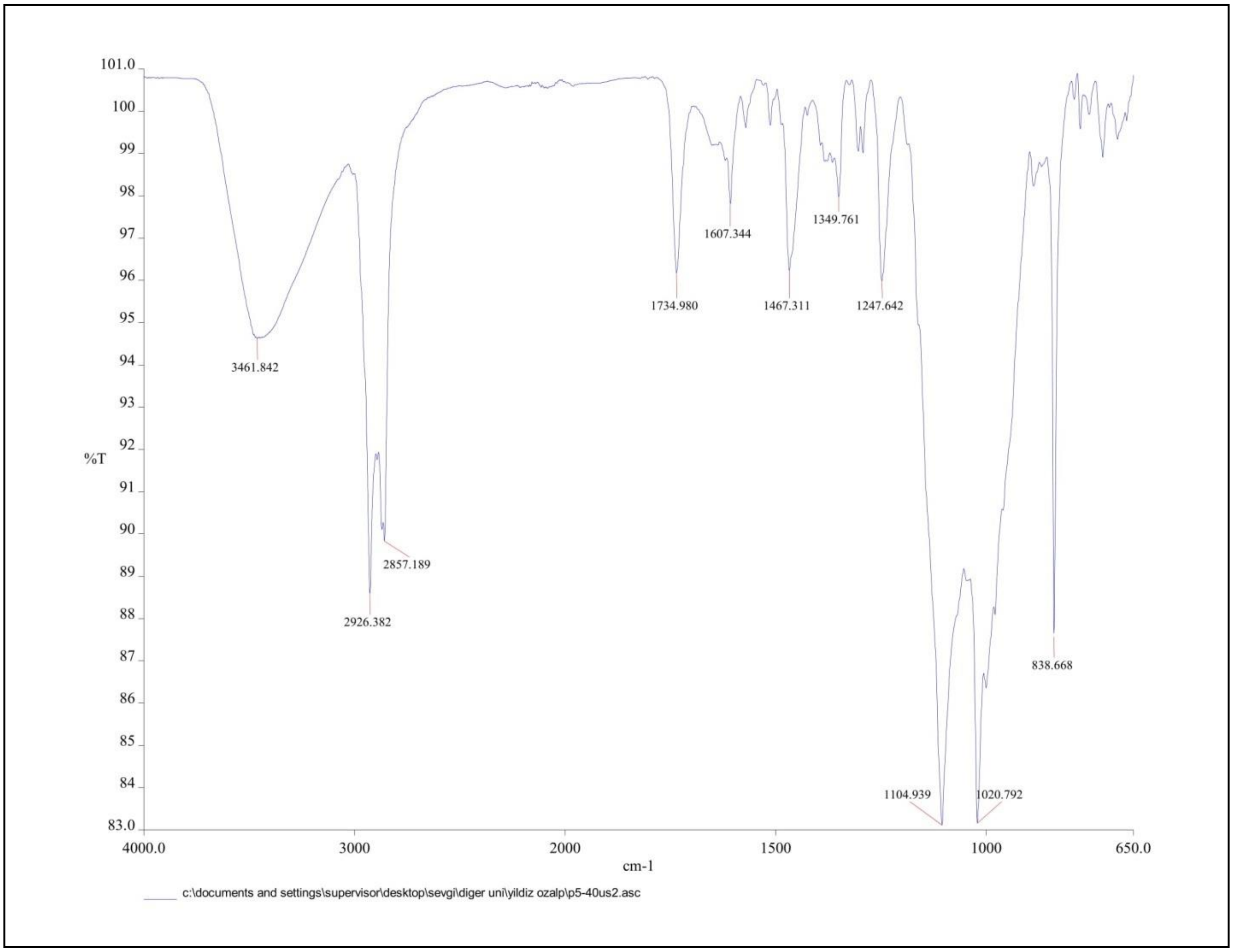

Figure S3. FT-IR spectrum of p5-50-us2 formulation.

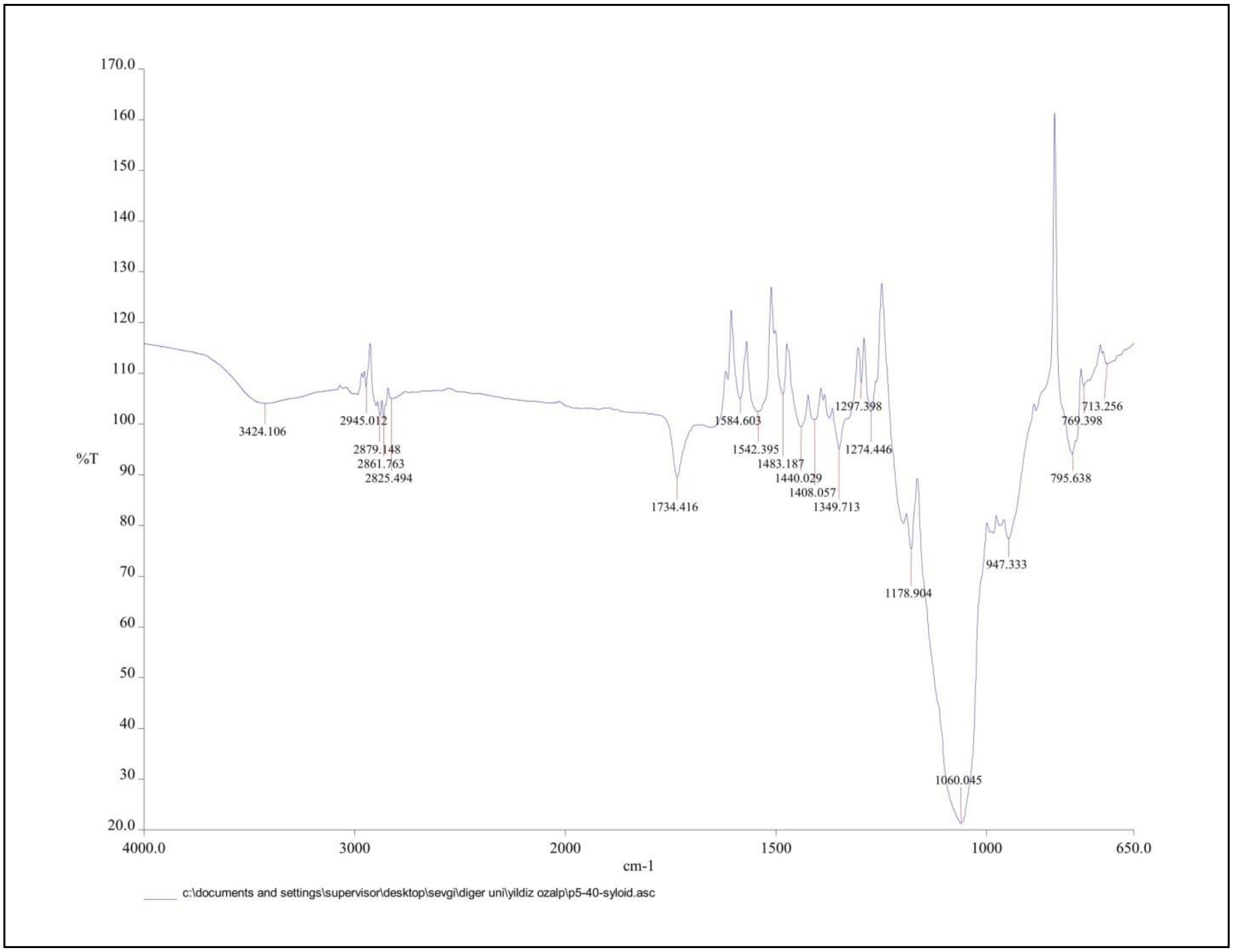

Figure S4. FT-IR spectrum of p5-40-syloid.

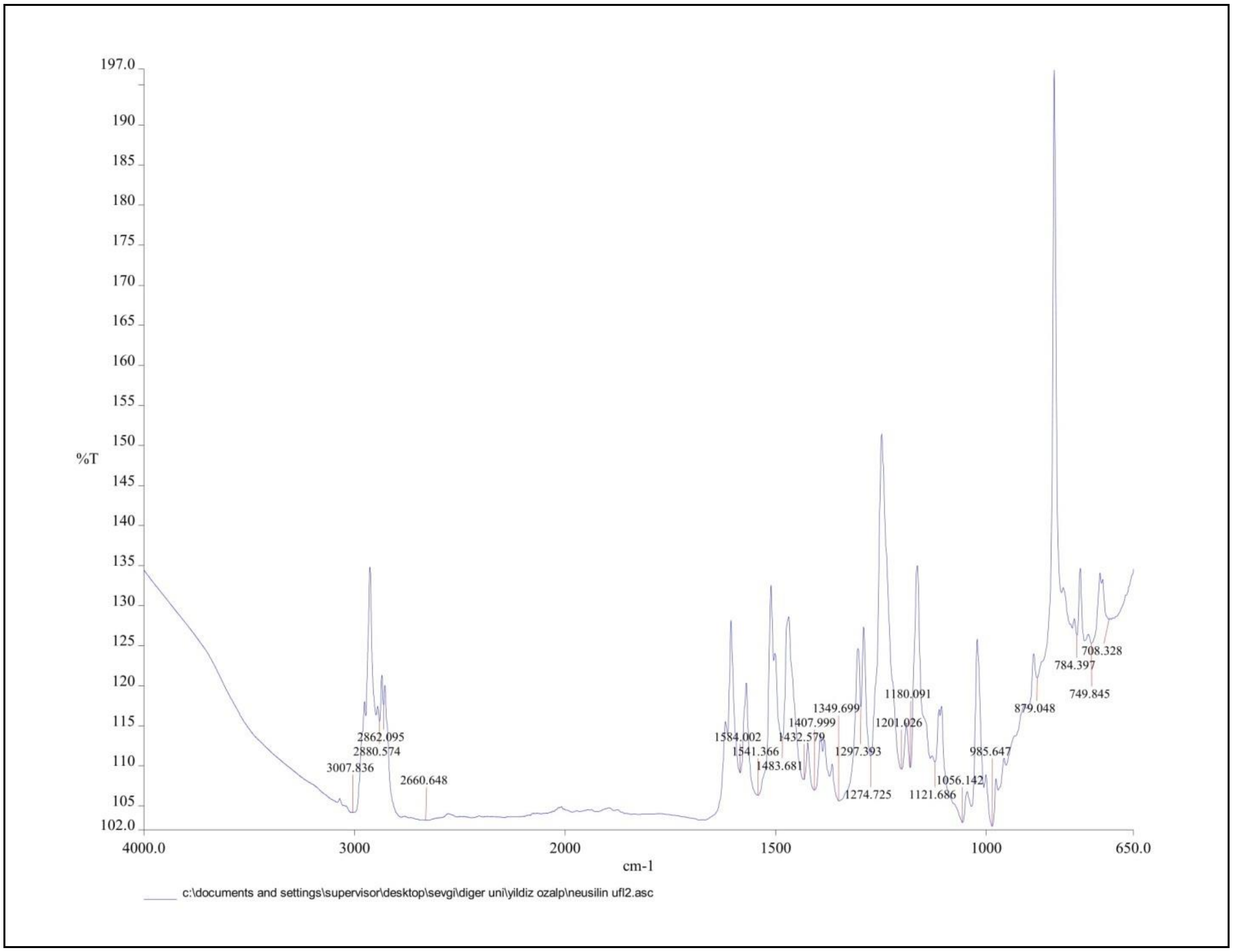

Figure S5. FT-IR spectrum of neusilin ufl2 carrier.

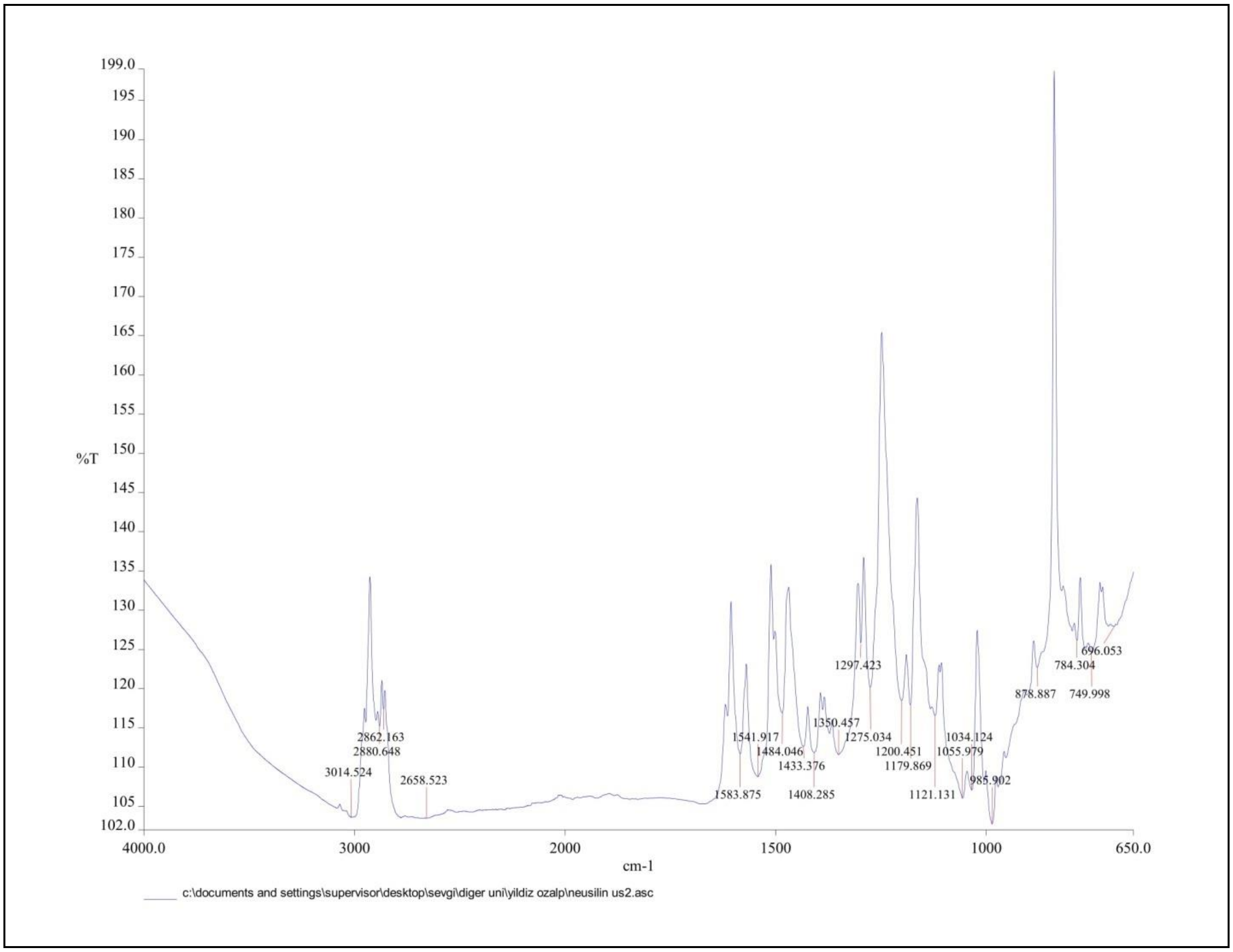

Figure S6. FT-IR spectrum of neusilin us2 carrier.

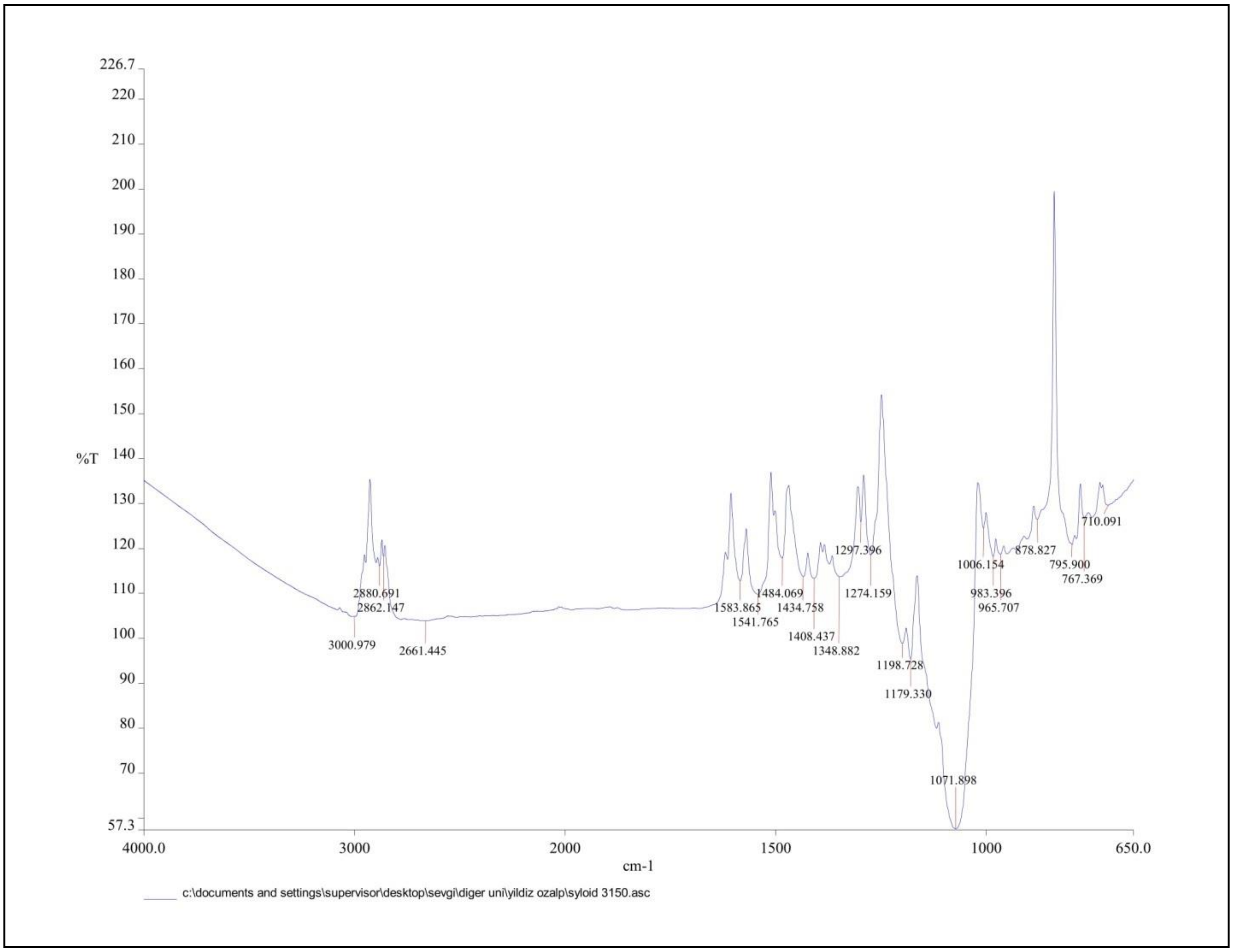

Figure S7. FT-IR spectrum of syloid xdp 3150 carrier.
